# Supplementary material for: Design principles of a microtubule polymerase
Source: eLife. 2018 Jun 13;7:e34574. doi: 10.7554/eLife.34574 (PMC5999394; doi:10.7554/eLife.34574)
Supplement: Transparent reporting form [file elife-34574-transrepform.docx]

***eLife’s* transparent reporting form**

We encourage authors to provide detailed information *within their submission* to facilitate the interpretation and replication of experiments. Authors can upload supporting documentation to indicate the use of appropriate reporting guidelines for health-related research (see [EQUATOR Network](http://www.equator-network.org/%20)), life science research (see the [BioSharing Information Resource](https://biosharing.org/)), or the [ARRIVE guidelines](http://www.plosbiology.org/article/info:doi/10.1371/journal.pbio.1000412) for reporting work involving animal research. Where applicable, authors should refer to any relevant reporting standards documents in this form.

If you have any questions, please consult our Journal Policies and/or contact us: [editorial@elifesciences.org](mailto:editorial@elifesciences.org).

**Sample-size estimation**

- You should state whether an appropriate sample size was computed when the study was being designed
- You should state the statistical method of sample size computation and any required assumptions
- If no explicit power analysis was used, you should describe how you decided what sample (replicate) size (number) to use

Please outline where this information can be found within the submission (e.g., sections or figure legends), or explain why this information doesn’t apply to your submission:

These concern/questions do not apply to our submission, which almost exclusively involves in vitro experiments using purified protein. Replicates and statistical analyses are indicated throughout, as described below.

**Replicates**

- You should report how often each experiment was performed
- You should include a definition of biological versus technical replication
- The data obtained should be provided and sufficient information should be provided to indicate the number of independent biological and/or technical replicates
- If you encountered any outliers, you should describe how these were handled
- Criteria for exclusion/inclusion of data should be clearly stated
- High-throughput sequence data should be uploaded before submission, with a private link for reviewers provided (these are available from both GEO and ArrayExpress)

Please outline where this information can be found within the submission (e.g., sections or figure legends), or explain why this information doesn’t apply to your submission:

We indicated replicates for each piece of data in the corresponding figure legend for all figures (1-6) and supplemental figures (1-1, 2-1, 4-1, 5-2). We did not encounter/exclude outliers, and the experiments reported do not involve high-throughput data of any kind.

**Statistical reporting**

- Statistical analysis methods should be described and justified
- Raw data should be presented in figures whenever informative to do so (typically when N per group is less than 10)
- For each experiment, you should identify the statistical tests used, exact values of N, definitions of center, methods of multiple test correction, and dispersion and precision measures (e.g., mean, median, SD, SEM, confidence intervals; and, for the major substantive results, a measure of effect size (e.g., Pearson's r, Cohen's d)
- Report exact p-values wherever possible alongside the summary statistics and 95% confidence intervals. These should be reported for all key questions and not only when the p-value is less than 0.05.

Please outline where this information can be found within the submission (e.g., sections or figure legends), or explain why this information doesn’t apply to your submission:

In each of the figure legends where data is presented, we explained how fits to the data were performed, the exact values of N we used for each, how many replicates for each set were performed, and the corresponding SEM error intervals that are shown. This information was provided in figure legends for Figures 1, 2, 3, 4 (replicates),5 (replicates) and 6, as well as in figure legends for Figure 1- figure supplement 1, Figure 2- figure supplement 1, and Figure 4- figure supplement 1.

Raw data was presented in multiple figures as images to show the corresponding phenotypes associated with statistical data. This includes raw images in Figures 1, 3, 4, 5 and 6, and Figure 1-figure supplement 1, Figure 2- figure supplement 1, Figure 5- figure supplements 1,2 . Figure 2A shows data as a ‘dot plot’ instead of providing summary statistics.

(For large datasets, or papers with a very large number of statistical tests, you may upload a single table file with tests, Ns, etc., with reference to sections in the manuscript.)

**Group allocation**

- Indicate how samples were allocated into experimental groups (in the case of clinical studies, please specify allocation to treatment method); if randomization was used, please also state if restricted randomization was applied
- Indicate if masking was used during group allocation, data collection and/or data analysis

Please outline where this information can be found within the submission (e.g., sections or figure legends), or explain why this information doesn’t apply to your submission:

Does not apply to our submission. Samples were not allocated into experimental groups.

**Additional data files (“source data”)**

- We encourage you to upload relevant additional data files, such as numerical data that are represented as a graph in a figure, or as a summary table
- Where provided, these should be in the most useful format, and they can be uploaded as “Source data” files linked to a main figure or table
- Include model definition files including the full list of parameters used
- Include code used for data analysis (e.g., R, MatLab)
- Avoid stating that data files are “available upon request”

Please indicate the figures or tables for which source data files have been provided:

We provided information on fit parameters to data in summary tables in Figures 1, 2, 3 and 6, as well as Figure 1-figure supplement 1, Figure 2-figure supplement 1, Figure 4- figure supplement 1 and Figure 5- figure supplement 1.

We have included numerical source data for Figures 1, 2, 3 and 6, and Figure 1- figure supplement 1, Figure 2- figure supplement 1, Figure 4-figure supplement 1. All numerical source data files are excel files.
